# Exploring the genes involved in biosynthesis of dihydroquercetin and dihydromyricetin in *Ampelopsis grossedentata*

**DOI:** 10.1038/s41598-021-95071-x

**Published:** 2021-08-02

**Authors:** Zheng-Wen Yu, Ni Zhang, Chun-Yan Jiang, Shao-Xiong Wu, Xia-Yu Feng, Xiao-Ying Feng

**Affiliations:** 1 School of Life Sciences, Guizhou Normal University, Guiyang, 550025 China; 2 State Key Laboratory of Functions and Applications of Medicinal Plants, Guizhou Medical University, Guiyang, 550014 China; 3 The Key Laboratory of Chemistry for Natural Products of Guizhou Province and Chinese Academy of Sciences, Guiyang, 550014 China

**Keywords:** Biochemistry, Transcriptomics

## Abstract

Dihydroquercetin (DHQ), an extremely low content compound (less than 3%) in plants, is an important component of dietary supplements and used as functional food for its antioxidant activity. Moreover, as downstream metabolites of DHQ, an extremely high content of dihydromyricetin (DHM) is up to 38.5% in *Ampelopsis grossedentata.* However, the mechanisms involved in the biosynthesis and regulation from DHQ to DHM in *A. grossedentata* remain unclear. In this study, a comparative transcriptome analysis of *A. grossedentata* containing extreme amounts of DHM was performed on the Illumina HiSeq 2000 sequencing platform. A total of 167,415,597 high-quality clean reads were obtained and assembled into 100,584 unigenes having an N50 value of 1489. Among these contigs, 57,016 (56.68%) were successfully annotated in seven public protein databases. From the differentially expressed gene (DEG) analysis, 926 DEGs were identified between the B group (low DHM: 210.31 mg/g) and D group (high DHM: 359.12 mg/g) libraries, including 446 up-regulated genes and 480 down-regulated genes (B vs. D). Flavonoids (DHQ, DHM)-related DEGs of ten structural enzyme genes, three myeloblastosis transcription factors (MYB TFs), one basic helix–loop–helix (bHLH) TF, and one WD40 domain-containing protein were obtained. The enzyme genes comprised three *PALs*, two *CLs*, two *CHSs*, one *F3’H*, one *F3’5’H* (directly converts DHQ to DHM), and one *ANS*. The expression profiles of randomly selected genes were consistent with the RNA-seq results. Our findings thus provide comprehensive gene expression resources for revealing the molecular mechanism from DHQ to DHM in *A. grossedentata*. Importantly, this work will spur further genetic studies about *A. grossedentata* and may eventually lead to genetic improvements of the DHQ content in this plant.

## Introduction

Dihydroquercetin (DHQ), also known as 3,5,7,3,4-pentahydroxy flavanone or commonly as taxifolin, is a kind of bioactive flavonoid. Because the molecular structure of DHQ contains five phenolic hydroxyl groups, it is considered to be one of the best and rarest natural powerful antioxidants in the world^1^. The safety of DHQ also has been investigated since it is a key component of dietary supplements^2^. Additionally, DHQ has a wide range of pharmacological activities, including anti-inflammatory^3^, anti-microbial^4^ anti-cancer^5^, anti-Alzheimer^6^, anti-toxoplasmosis effects^7^, health-promoting effects on hepatoprotective and cardiovascular systems^8, 9^. Due to its excellent pharmacological activity, DHQ is routinely used in pharmaceuticals, health products, foods and agriculture.

DHQ is most prevalent in larch, Douglas fir bark, French maritime pine bark, milk thistle, and onions^2, 10–13^. Among them, larch is the plant with the highest content (about 3%) of DHQ^2, 14^, but its distribution is sporadic, limited in range to a few countries, namely China, Japan, and Russia. Recently, DHQ has been obtained with low yield from plant, thereby limiting its widespread applications. It is less than annual output of 20 tons in global. Among them, China currently only produces around 5 tons. Unfortunately, many plants not only have a low content of DHM, but also upstream compounds (eriodictyol, dihydrokaempfrol) and downstream compounds (dihydromyricetin, quercetin, leucocyanidin) involved in the biosynthesis of DHQ likewise occur in low amounts^15, 16^. Consequently, it is very difficult to enhance the content of DHQ in these plants via conventional genetic techniques for improvement.

*Ampelopsis grossedentata*, a member of the Vitaceae plant family, grows widely in mountainous areas of southern China, and contains a high content of dihydromyricetin (DHM) in immature leaves: over 20% in dry leaves of most individuals and up to 38.5% in a few less common ones^17–19^. Interestingly, the plant contains a low content of DHQ, which is a direct precursor of DHM’s biosynthesis^20, 21^*.* DHM shares a similar molecular structure with DHQ, the former converted from DHQ by adding a phenolic hydroxyl under the catalysis of special enzymes. Therefore, genetic manipulation of such related metabolic pathways is one useful strategy to improve the yield of DHQ. However, the candidate genes involved in flavonoid (DHQ and DHM) biosynthesis and regulation in *A. grossedentata* remain unclear.

The enzymes and related genes involved in flavonoid biosynthesis and regulation have been reported in many plants, such as *Phyllanthus emblica (L.)*^22^, *Ginkgo biloba*^23^, *Semen Trigonellae*^24^, *Meconopsis*^25^, *Arabidopsis thaliana*^26^, *Mangifera indica*^27^, *Salvia miltiorrhiza*^28^and *A. grossedentata*^29^. Several enzymes in the biosynthetic pathway of flavonoids have been studied, including phenylalanine ammonia lyase (PAL), cinnamate 4-hydroxylase (C4H), 4-coumaroyl-CoA ligase (4CL), chalcone synthase (CHS), and chalcone isomerase (CHI), which are known to catalyze naringenin synthesis from L-phenylalanine (L-Phe). Subsequently, flavanone 3-hydroxylase (F3H), flavonoid 3’-hydroxylase (F3’H), flavonoid-3’,5’-hydroxylase (F3’5’H), dihydroflavonol-4-reducatse (DFR), flavonol synthase (FLS), and anthocyanidin synthase (ANS) are responsible for the later steps in the synthesis pathway^30^. Flavonoid biosynthesis is regulated by myeloblastosis transcription factors (MYB TFs), basic helix–loop–helix (bHLH) TFs, and WD40 proteins^31^. Among them, AtMYB12 has been identified as the main regulator of phenylpropanoid biosynthesis that up-regulates the expression of *CHS*, *CHI*, *F3H*, and *FLS*^32^.

In order to illustrate the physiological, biochemical and environmental factors for accumulation of dihydromyricetin, the effects of PAL and CHI on the metabolism of dihydromyricetin under different soil conditions were studied and analyzed in our research^33, 34^. RNA sequencing (RNA-Seq) has been applied in transcriptome studies of *A. grossedentata* in different tissues and leaf stages, the study just showed the expression patterns and differential distribution of genes related to DHM bisosynthesis in *A. grossedentata*^29^. However, there have been no studies investigating and discussing the molecular mechanisms of flavonoid (DHQ and DHM) formation and accumulation in *A. grossedentata* with same developmental period, different genetic backgrounds. In this study, *A. grossedentata* containing an extreme content of DHM were chosen as the experimental material. The candidate genes involved in the flavonoid (DHQ and DHM) biosynthesis and regulation pathways in *A. grossedentata* was analyzed and investigated by the comparative transcriptome, especially the transformation relationship between DHQ and DHM. The obtained transcriptome data will thus serve as reference sequences for genetic studies of *A. grossedentata* in the future. Additionally, this study provides useful resources for further study of the transformation from DHQ to DHM, and plays crucial roles for revealing the DHQ’s formation mechanism in *A. grossedentata*.

## Materials and methods

### Plant materials

**Figure 1:**
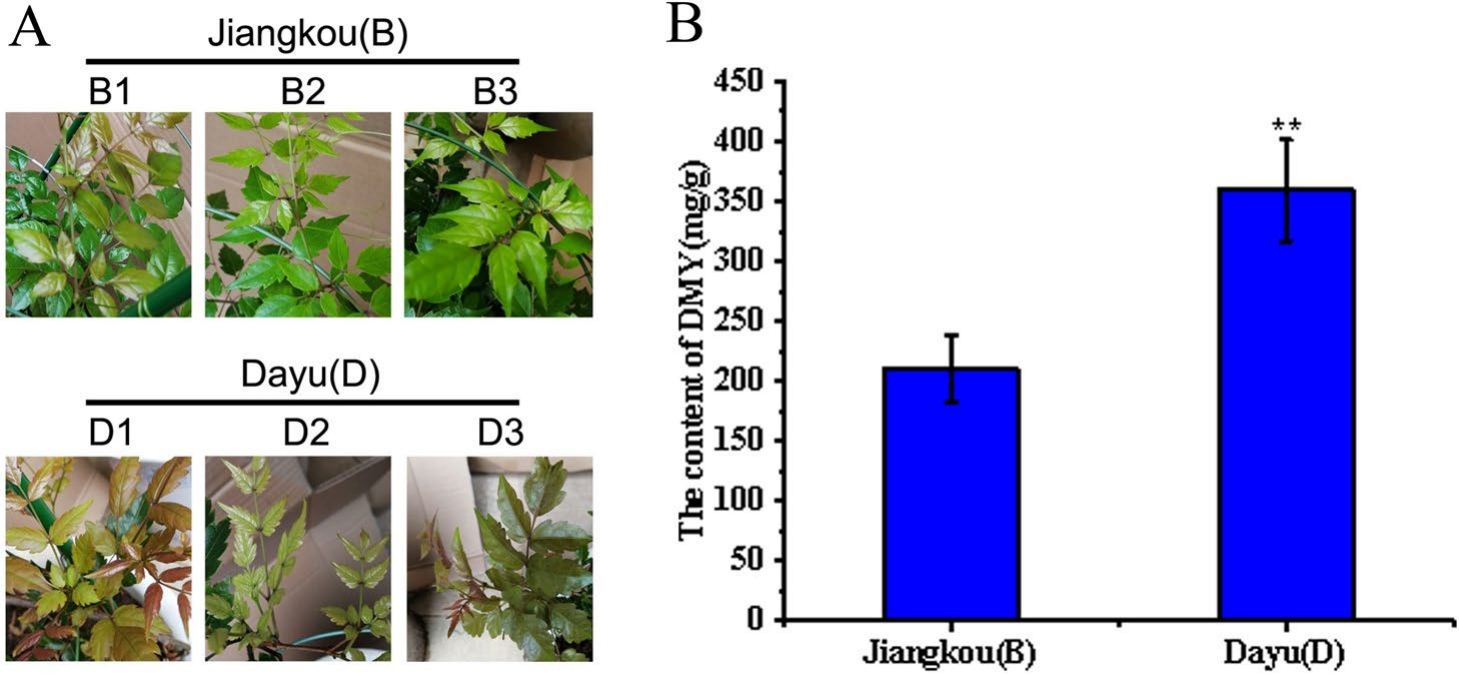
The difference of DHM content of *A. grossedentata* in two groups: **(A)** Phenotypic characteristic of *A. grossedentata* immature leaves used for RNA-seq. **(B)** HPLC analysis of the DHM content of *A. grossedentata* in B and D groups. Values indicate the mean ± SD. The double asterisk indicates statistically significantly different means from the B group (Student’s t-test, P < 0.01).

*A. grossedentata* was collected from Dayu of Jiangxi Province (No. D1–D3, three independent individuals) and Jiangkou of Guizhou Province (No. B1–B3, three independent individuals) in 2012. The collected scions were preserved in the form of sapling and planted in the *A. grossedentata* germplasm resource repository of Guizhou Normal University (26°26′18.23″ N, 106°39′45.32″ E, at 1100.5 m a.s.l.) (Guiyang, Guizhou Province, China). The plant was identified by Associate Prof. Chao Zhang of School of Life Sciences, Guizhou Normal University. The voucher specimens (accession number: GZNUYZW202002001) was deposited in the herbarium of School of Life Sciences, Guizhou Normal University. For comparative transcriptome analysis, same-aged individuals of B1, B2, B3, D1, D2 and D3 were cultivated closely and under the same management practices (consistent light, soil, and moisture conditions) (Fig. 1A). In May 2018, their young leaf samples were frozen in liquid nitrogen and stored at –80°C until later use.

### HPLC analysis of the DHM and its biosynthesis related compounds

An ultrasonic method to extract total flavonoids from leaves was used. Approximately 0.1 g of oven-dried leaf tissue was weighed per sample and placed into a centrifuge tube, to which 10 mL of methanol extract was added. Ultrasonic extraction was performed at 30 °C for 30 min followed by centrifugation at 12,000 rpm at 25 °C for 10 min; 5 mL of the ensuing supernatant was used for further analyses. The content of DHM, DHQ, Myricetin, Myricetrin ware measured by high performance liquid chromatography (HPLC), which used methanol: acetonitrile (5:11) as solvent A (0.1% formic acid as solvent B) 0–10 min, 20:80, 11–20 min, 40:60, 21–25 min, 20:80. The column temperature was 25 °C, the sample volume injected was 20.0 µL, and the flow rate was 1.0 mL/min, with the detector set to 325 nm.

The DHM determination results of this study generated two groups of plant material—one with a relatively high DHM content (D group, Fig. 1B) and the other with a relatively low DHM content (B group, Fig. 1B)—being selected for the transcriptome sequencing. Each group had three independent individuals (i.e., three biological replicates).

### RNA sequencing(RNA-seq)and bioformatics analysis

#### RNA extraction

The total RNA was extracted from young leaf samples in D and B groups using a mirVana miRNA Isolation Kit (Ambion) by following the manufacturer’s protocol as described in previous paper^35^. Total RNA purity and concentration of each sample were measured using the NanoDrop 2000 (Thermo Scientific, Wilmington, DE, USA), and RNA integrity then assessed with the RNA Nano 6000 Assay Kit of the Agilent Bioanalyzer 2100 system (Agilent Technologies, Santa Clara, CA, USA). Only those samples with an RNA integrity number (RIN) ≥ 7 were retained for subsequent analysis^36^.

#### Library construction and sequencing

A total amount of 1μg RNA per sample was used as input material for the RNA sample preparations. Sequencing libraries were constructed using the NEBNext Ultra RNA Library Prep Kit for Illumina (NEB, USA) following the manufacturer’s recommendations, for which index codes were added to attribute sequences to each sample^37^. The clustering of these index-coded samples was performed on a cBot Cluster Generation System, using the TruSeq PE Cluster Kit v3-cBot-HS (Illumina) according to the manufacturer’s instructions. Subsequently, the library preparations were sequenced on an Illumina Hiseq 2000 platform and the paired-end reads were generated^38^. The sequencing data have been deposited in the NCBI Sequence Read Archive (SRA), and are accessible through the accession number PRJNA736327.

### De novo assembly and functional annotation

Raw data (i.e., raw reads) were processed through in-house perl scripts in FastQC software (http://www.bioinformatics.babraham.ac.uk/projects/fastqc/), with low-quality reads were discarded using the NGS QC Toolkit (v2.3.3) software program (http://59.163.192.90:8080/ngsqctoolkit/). After reads adapter, reads containing ploy-N and low-quality reads from the raw data were removed, leaving only clean reads that were then assembled into transcripts de novo, by using the Trinity v2.5.1 software (https://github.com/trinityrnaseq/trinityrnaseq/wiki) as well as the paired-end method with min kmer cov set to 2 (by default) and all other parameters at their default settings^39^. The longest transcript was designated a unigene based on the similarity and length of a sequence for subsequent analysis.

The function of a given unigene was annotated by alignment of the unigenes with the seven following databases: NCBI non-redundant protein sequences (NR); Pfam (Protein family); KOG/COG (Clusters of Orthologous Groups of proteins); Swiss-Prot (a manually annotated and reviewed protein sequence database); KEGG (Kyoto Encyclopedia of Genes and Genomes); GO (Gene Ontology).

### Expression annotation and enrichment analysis for differentially expressed genes (DEGs)

Relative gene expression levels were estimated by using the RSEM (RNA-Seq by expectation maximization) method for each sample^40^. We used the fragments per kilobase per million reads (FPKM) method to calculate the expression abundance of each unigene obtained. Differential expression analysis of two conditions/groups was performed with the DESeq R package (http://www.bioconductor.org/packages/release/bioc/html/DESeq.html). DESeq provide statistical routines for testing the differential expression via a negative binomial distribution and a shrinkage estimator for the variance of the distribution; the resulting P values were then adjusted using the Benjamini-Hochberg adjustment for controlling the false discovery rate (FDR). The FDR served as a threshold of the P-value in multiple tests, to reliably judge the significance of gene expression differences. From this, DEGs had to satisfy both criteria of an FDR < 0.01 and a fold change (FC ≥ 2) threshold value. A hierarchical cluster analysis of DEGs was done to explore the patterns of gene expression^41^. GO enrichment analysis of the DEGs was implemented by the topGO (v2.28.0) R package-based (http://www.bioconductor.org/packages/release/bioc/html/topGO.html) Kolmogorov–Smirnov test. The Kyoto Encyclopedia of Genes and Genomes (KEGG) pathway enrichment analysis is a database resource for understanding high-level functions and utilities of biological systems^42^. We used KOBAS software (KOBAS v2.0, http://kobas.cbi.pku.edu.cn/help.do) to test the statistical enrichment of DEGS in KEGG pathways^43^.

### Quantitative real-time PCR (QRT-PCR) analysis of gene expression

Total RNA extraction and the qRT-PCR were carried out following a published protocol^44^. The putative *glyceraldehyde-3-phosphate dehydrogenase* (*GAPDH*) gene of *A. grossedentata*, annotated in our transcriptome database (Gene ID, c53602.graph_c2), served as the reference gene for qRT-PCR. These gene-specific primers were used: 1F and 1R for *AgF3’H* (Gene ID, c48392.graph_c0); 2F and 2R for *AgCHS* (Gene ID, c52193.graph_c2); 3F and 3R for *AgF3’5’H* (Gene ID, c52962.graph_c0); 4F and 4R for *AgANS* (Gene ID, c53486.graph_c2), and; 5F and 5R for *AgGAPDH*; Table S1).

### Statistical analysis

Statistical analysis was performed in SPSS 19.0 software. Statistical significance (P < 0.05; P < 0.01) was assessed by using the student's *t*-test.

## Results

### Determination of DHM, DHQ, myricetin and myricetrin levels of B and D groups

Here we evaluated two groups of plants from different geographical locations: the B group collected from Jiangkou of Guizhou Province (No. B1-B3, three independent individuals) and the D group collected from Dayu of Jiangxi Province (No. D1-D3, three independent individuals) (Fig. 1A). The DHM content of each group was measured by HPLC, and found significantly higher in the D group compared with the B group (Fig. 1B). Meanwhile, DHM, Myricetin and Myricetrin were determined to use analyse correlation between the content of four compounds. The result showed that DHM and DHQ, have a significant correlation (Table S2, Table S3). Based on these differing amounts of DHM, both groups were sequenced using the high-throughput Illumina HiSeq 2000 platform.

### Transcriptome sequencing and assembly

After adaptor sequences, unknown sequences, and any ambiguous and low-quality reads were removed from the raw data for the six replicate libraries, a mean of 28372227 clean reads from B group and 27432972 from D group were obtained (Table S4). More than 93.90% or 93.80% of the clean reads from B or D group had a Q-value > 30, and the GC base contents (%) were 46.24/46.45/47.30% and 46.35/46.24/46.12% respectively for B1/2/3 and D1/2/3 (Table S4). The assembly generated 100584 unigenes whose N50 length was 1489 bp and averaged 813.69 bp in length (Table S5). Of these unigenes, 26166 (26.01%) were 200 bp to 300 bp, 31061 (30.88%) were ranged from 300 bp to 500 bp, 21652(21.53%) were 500 bp to 1 kb, 11460 (11.39%) were 1 kb to 2 kb in length, and the remaining 10245 (10.19%) exceeded 2 kb (Table S5).

### Gene annotation and functional classification

All 100584 assembled unigenes were aligned to six protein databases using the BLAST algorithm with an E-value threshold of 10^−5^ (*E*-value ≤ 1.0 e^−5^) and to Pfam by using HMMER (*E*-value ≤ 1.0 e^−10^). In all, 57016 of these unigenes (56.68%) were successfully annotated (Table S6) as follows: 56,003 (98.22%) unigenes had significant matches in the Nr database, for which 17,392 were longer than 1000 bp; 35,776 (62.75%) unigenes were matched up in the Swiss-Prot database (12,531; length ≥ 1000 bp); 34,365 (60.27%) unigenes were significant matched in the Pfam; 21,197 (37.18%) unigenes showed high homology with sequences in the KEGG database; 42,682 (74.86%) could be annotated in the GO database, as were 29,881 (52.41%) in KOG and 14,876 (26.09%) in COG (Table S6).

**Figure 2:**
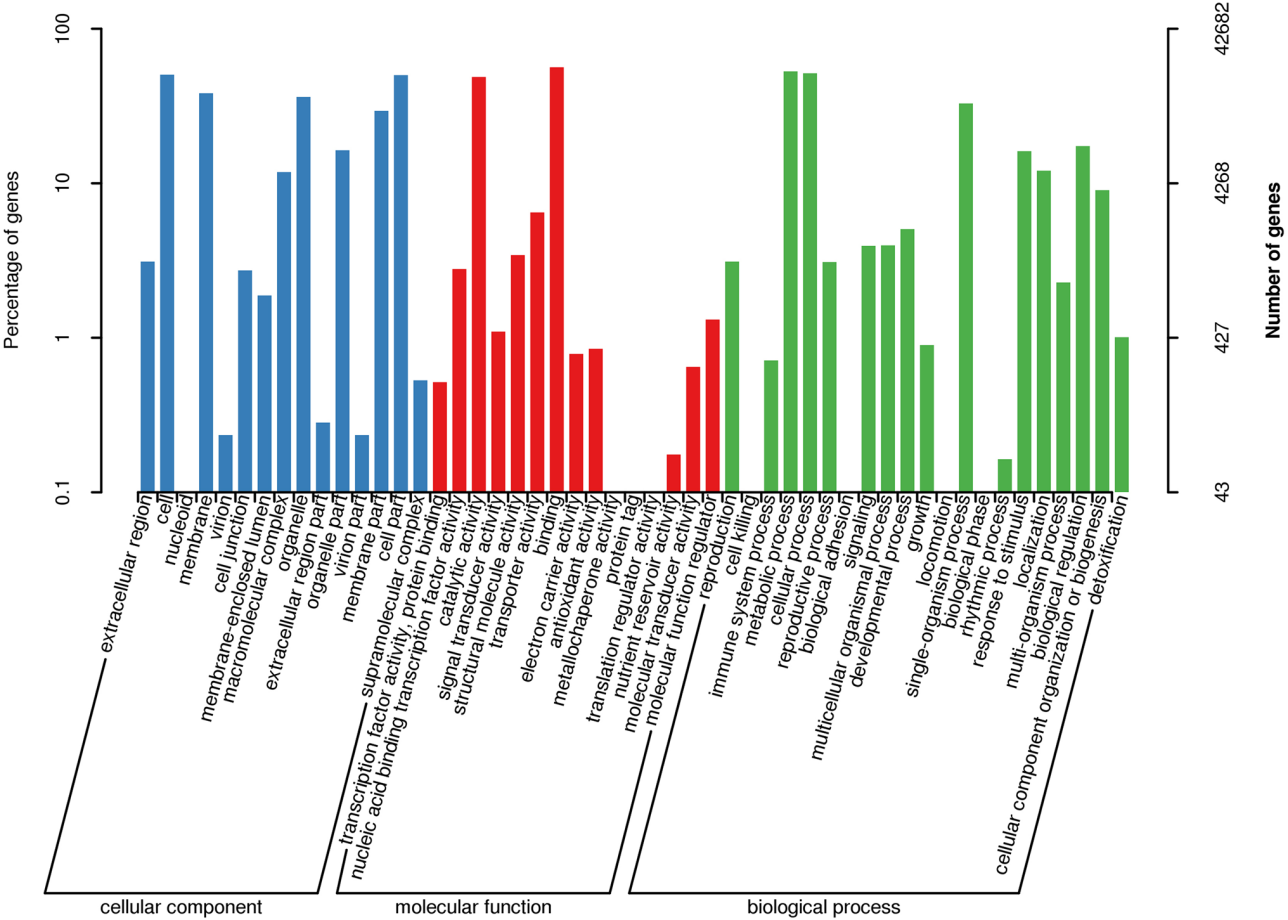
Gene Ontology (GO) classification of unigenes. All the annotated unigenes were divided into three functional GO categories: cellular component (CC), molecular function (MF), and biological process (BP). The x-axis represents the enriched GO terms. Left y-axis shows the percentage of unigenes in subcategories of each main category. Right y-axis indicates the number of unigenes in each subcategory.

Based on sequence similarity, all 42,682 unigenes in GO were assigned to its three main GO categories: cellular component (CC), molecular function (MF), and biological process (BP) (Fig. 2). In the CC subcategories (clustered into 15 GO terms), “cell” (21,489) and “cell part” (21,418) were the two highest-ranked subcategories (Fig. 2). In the MF subcategories, matched unigenes were also annotated to 15 GO terms, the two largest subcategories being “catalytic activity” (20,779) and “binding” (23,979) (Fig. 2). In the MF category (21 GO terms), the subcategory with the most unigenes assigned to it was “metabolic process” (22,587), the second-most being “cellular process” (22,047) (Fig. 2); these two subcategories indicated some important metabolic pathways operate in *A. grossedentata*, including that of flavonoid (DHQ and DHM) biosynthesis.

**Figure 3:**
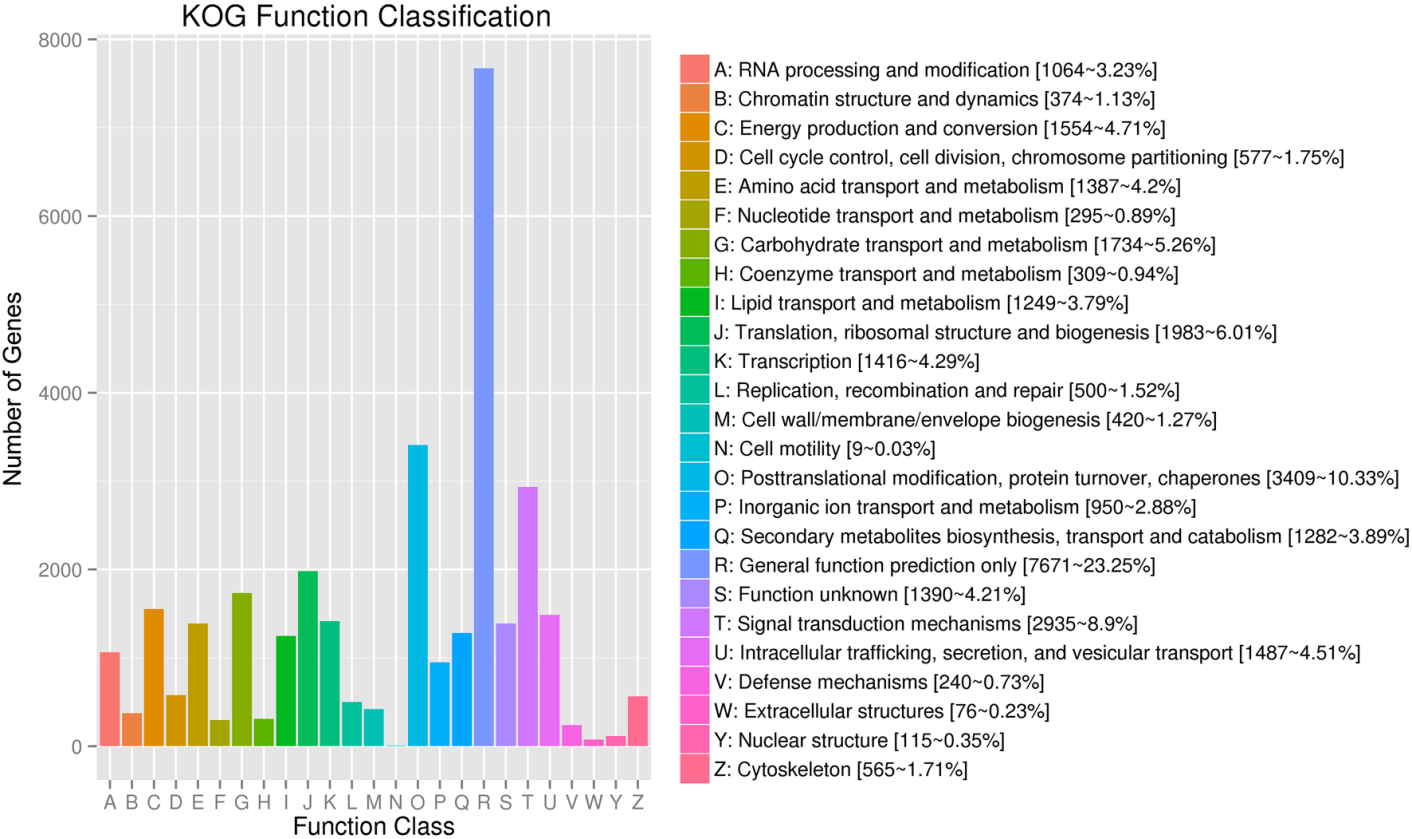
EuKaryotic clusters of Orthologous Groups (KOG) functional classification of unigenes. A total of 298, 81 annotated unigenes were found with significant homology in the KOG database (E-value ≤ 1.0 e^−5^). The x-axis indicates the names of the 25 KOG groups. The y-axis indicates the number of genes in 25 KOG categories. The numbers represent the count and percentage of unigenes in each category.

To further appraise the utility of the annotation process, we searched the annotated unigenes in KOG database and assigned 29,881 unigenes to the KOG terms (Table S6; Fig. 3). In the 25 KOG categories, the cluster for “General function prediction only” (7671, 23.25%) represented the largest category, followed by “Posttranslational modification, protein turnover, chaperones” (3409, 10.33%), “Signal transduction mechanisms” (2935, 8.9%), and “Translation, ribosomal structure and biogenesis” (1983, 6.01%). The “Secondary metabolites biosynthesis, transport and catabolism” group related to flavonoid biosynthesis accounted for just 3.89% (1282) of the sequences.

### Differentially expressed genes (DEGs) identification and enrichment (GO and KEGG) analyses

**Figure 4:**
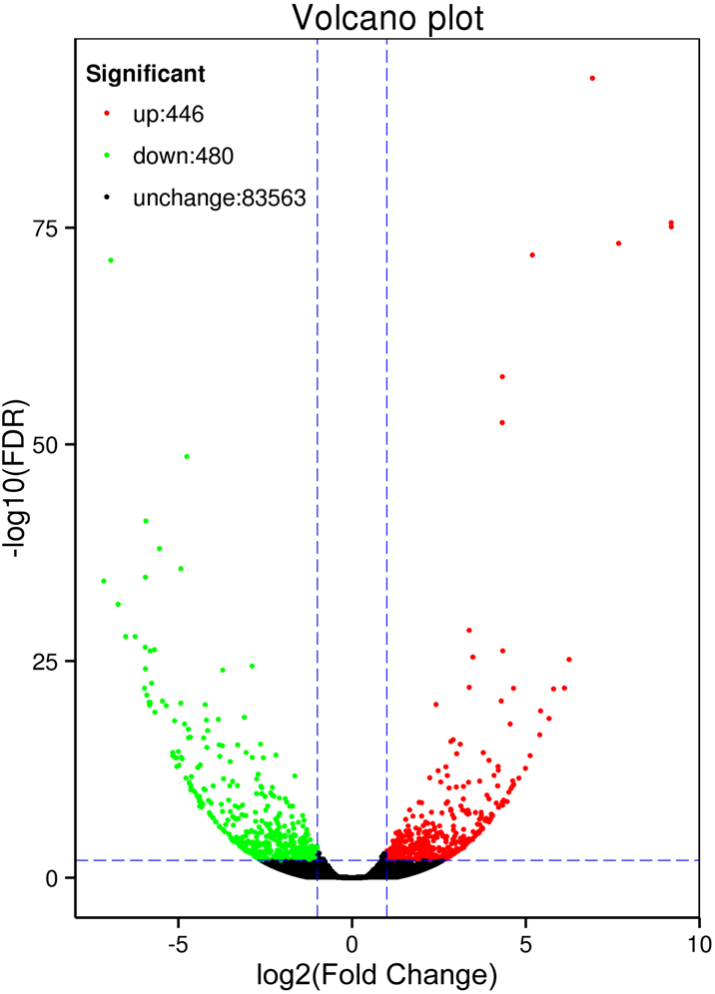
Expression patterns of unigenes between the B and D groups of plants. All 926 unigenes were identified as being differentially expressed (FDR < 0.01 and log2 (fold change) ≥ 2), including 448 up-regulated and 480 down-regulated genes. The red and green dots represent differentially expressed genes; the black dots indicate unchanged unigenes.

**Figure 5:**
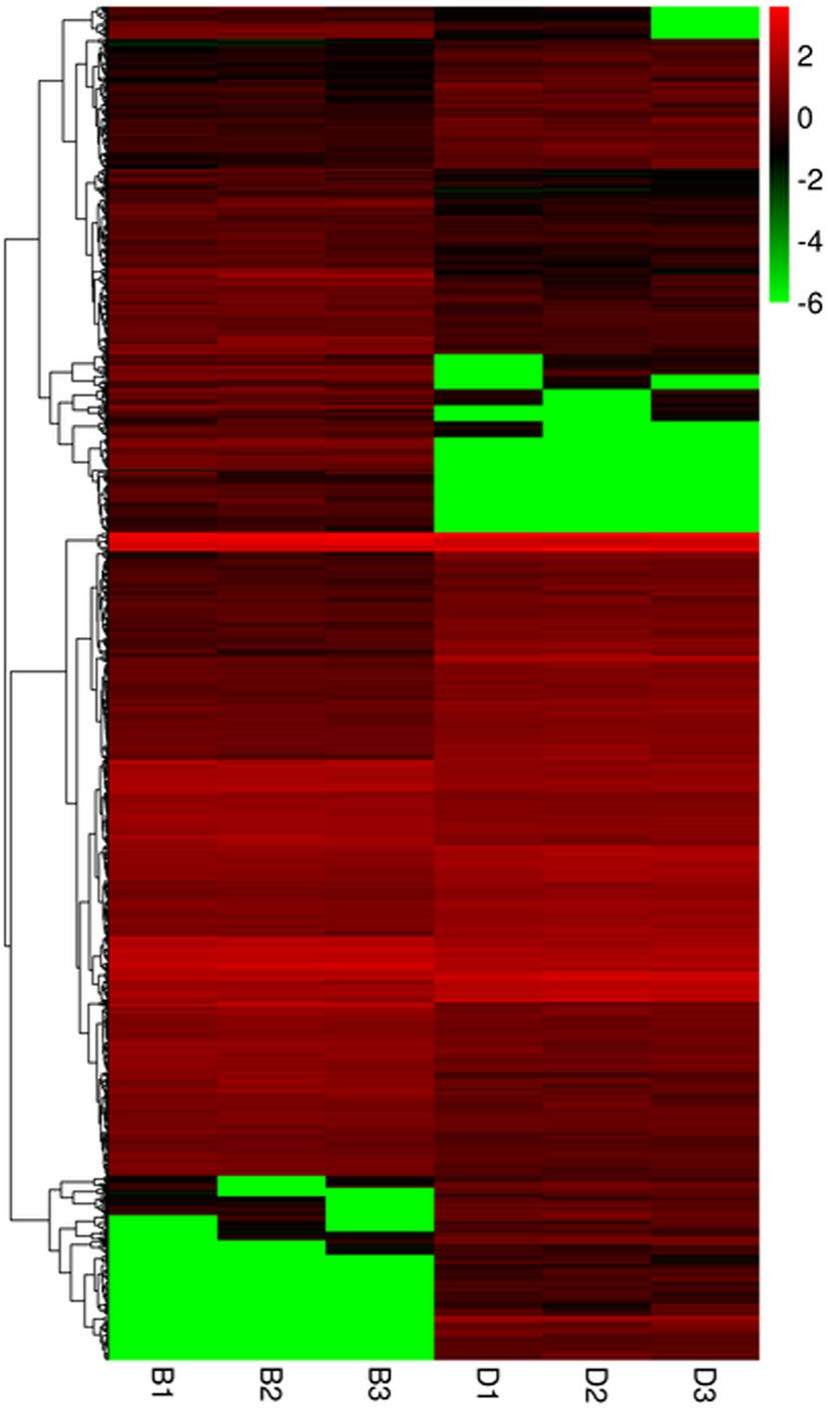
Heat map of differentially expressed genes based on a hierarchical cluster analysis. Green represents the lowest expression level; black indicates intermediate expression levels and red represents the highest expression levels. A color scale bar for log-transformed FPKM is shown at the top-right of the figure. B1, B2, and B3 represent three biological replicates from the B plants; likewise, D1, D2, and D3 are biological replicates from the D plants.

For 926 DEGs, their expression was identified as significantly changed between the B and D libraries, consisting of 446 up-regulated and 480 down-regulated genes (B vs. D) (Fig. 4). The clustering of DEGs indicated their relative abundance, by integrating the FPKM with coloring, in that red and green corresponded to relatively high and low expression levels, respectively (Fig. 5). The up-regulated DEGs are those with a color change from green to red. By comparing the expression profiles of the two plant groups, B1 was similar in color to B2/3, and all were classified into the same cluster. Many DEGs showed a relatively high expression level in the D1/2/3 heat map and displayed similar coloring (Fig. 5), which suggested strong agreement among those three independent biological replicates. All 926 DEGs were aligned to the seven protein databases, of 699 DEGs in total were annotated. Of these, 249 could be matched in the COG database, and likewise 523 in GO, 189 in KEGG, 355 in KOG, 486 in Pfam, 484 in Swiss-Prot database, and 696 in Nr. The details of the 699 annotated DEGs can be found in Table S7.

**Figure 6:**
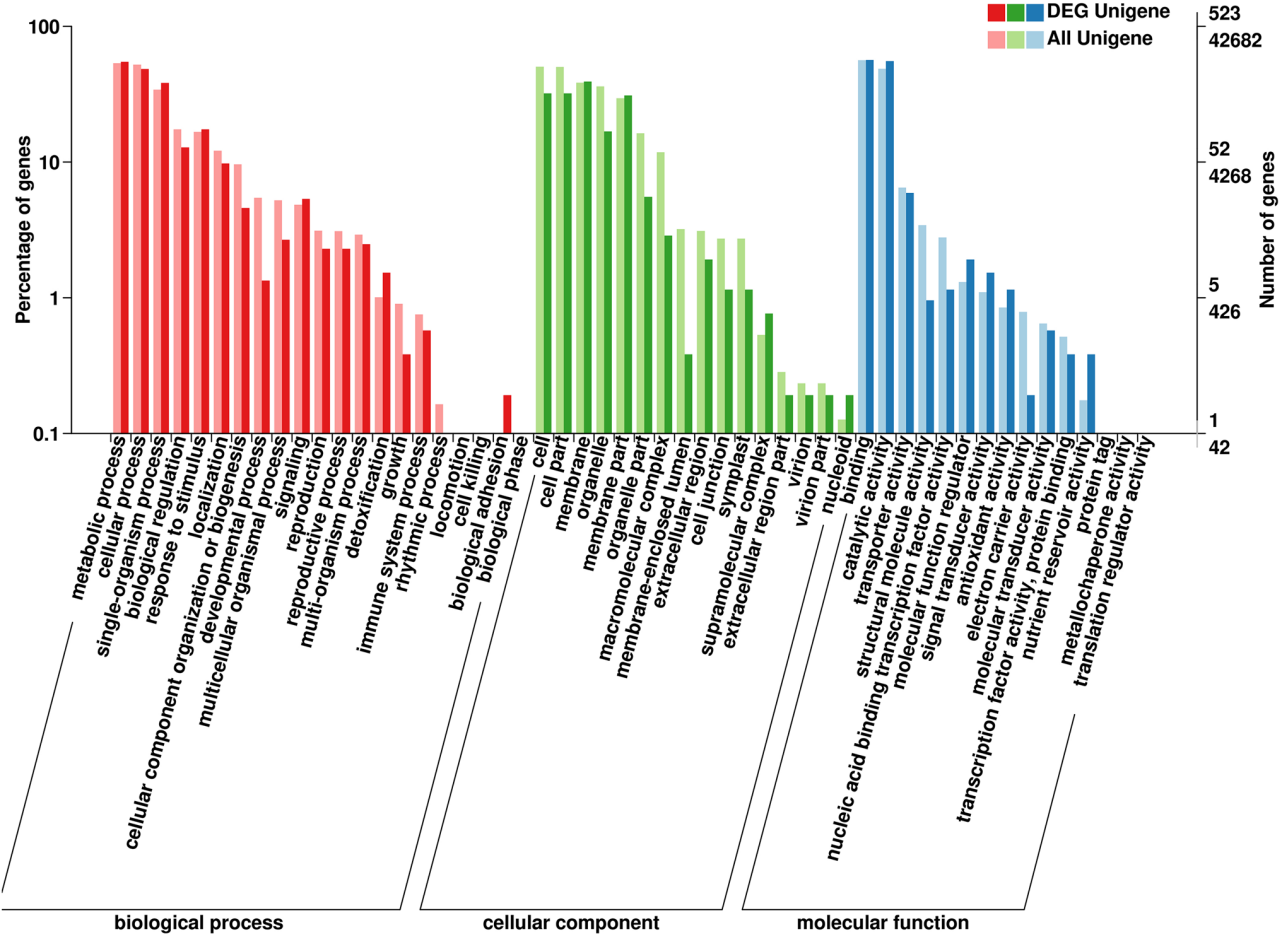
Functional GO classification of total annotated unigenes and DEGs. All annotated genes and DEGs were divided into three functional GO categories: biological process (BP), cellular component (CC), and molecular function (MF). The x-axis represents the enriched GO terms. The y-axis represents the number and percentage of genes.

All 523 DEGs associated with one or more GO terms were functionally annotated in GO database and assigned its three main categories (BP, CC, MF). In the BP category, 286, 253, and 200 DEGs were respectively classified into metabolic processes, cellular processes, and single-organism processes (Fig. 6); In the CC category, “cell” (168 DEGs) and “cell part” (168 DEGs) had a similar, high degree of enrichment, and the matched DEGs were classified into 13 GO terms of which the top ranked were “binding” (296 DEGs) and “catalytic activity” (200 DEGs); the “nucleic acid binding transcription factor activity” term, which is related to the regulation of flavonoid (DHQ and DHM) biosynthesis, contained 6 DEGs (Fig. 6).

**Figure 7:**
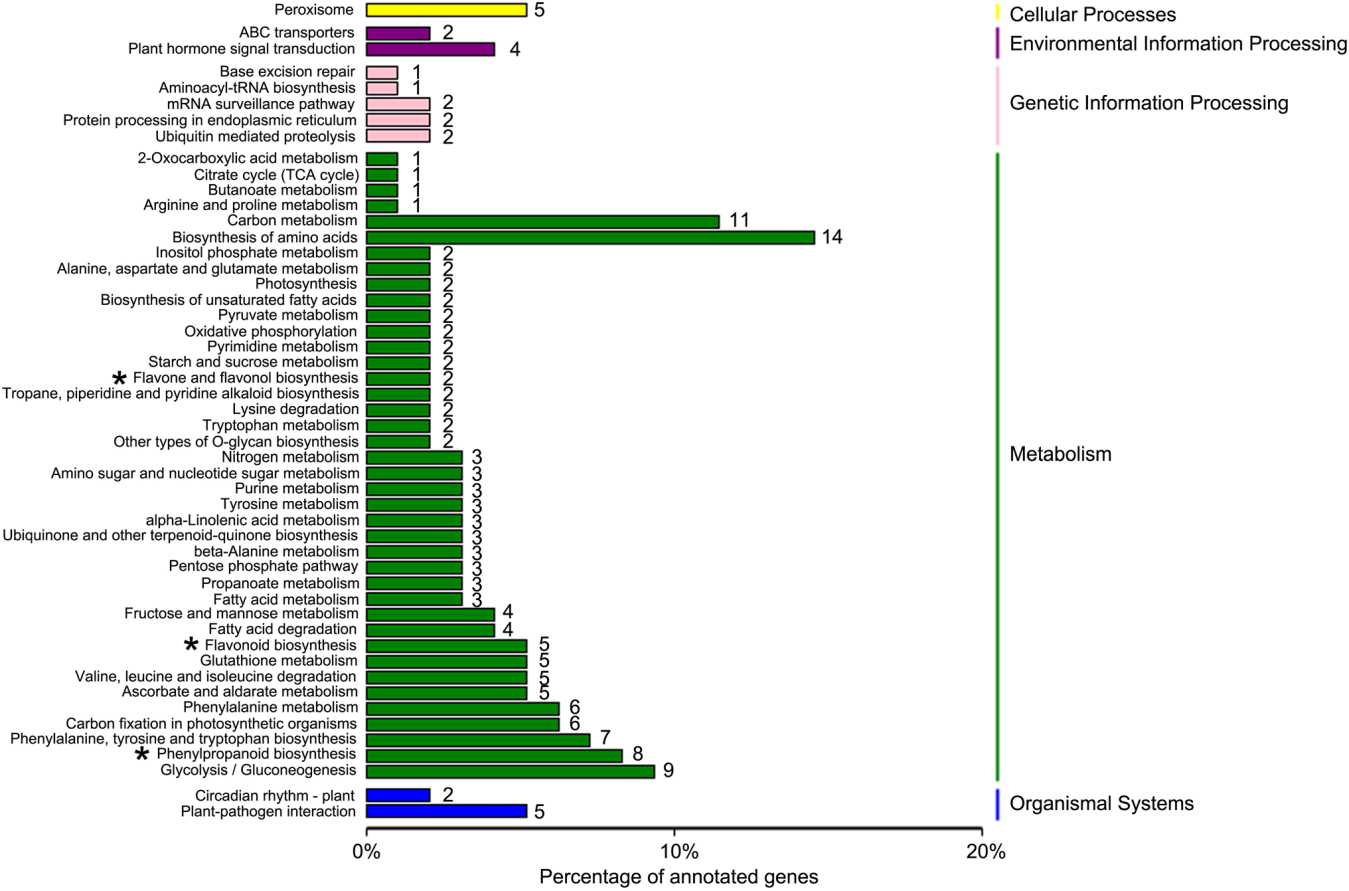
Kyoto Encyclopedia of Genes and Genomes (KEGG) pathway classification of differentially expressed genes (DEGs). The x-axis shows the proportion (%) of the total number of DEGs annotated that were annotated to a given pathway. The y-axis indicates each KEGG metabolic pathway name. The numbers indicate the number of DEGs annotated to each pathway. Single asterisks represent those pathways related to flavonoid biosynthesis.

All 926 DEGs were analyzed against the KEGG database, to which 189 DEGs were classified into 50 metabolic pathways (Fig. 7). These 50 pathways were assigned to five branches: cellular processes (5 DEGs), environmental information processing (6 DEGs), genetic information processing (8 DEGs), metabolism (163 DEGs), and organismal systems (7 DEGs) (Fig. 7). In the metabolism term, the top two pathways identified were “biosynthesis of amino acids (14)” and “carbon metabolism (11)” (Fig. 7). The 20 KEGG pathways with the highest representation of DEGs are depicted in Figure 7, for which the most significantly enriched pathway was “flavone and flavonol biosynthesis pathway.” The above KEGG annotations provided a useful resource for searching the metabolism pathways and processes involved in flavonoid (DHQ and DHM) biosynthesis in *A. grossedentata*.

### Identification of DEGs and candidate genes involved in the flavonoid biosynthesis and regulation pathways

**Figure 8:**
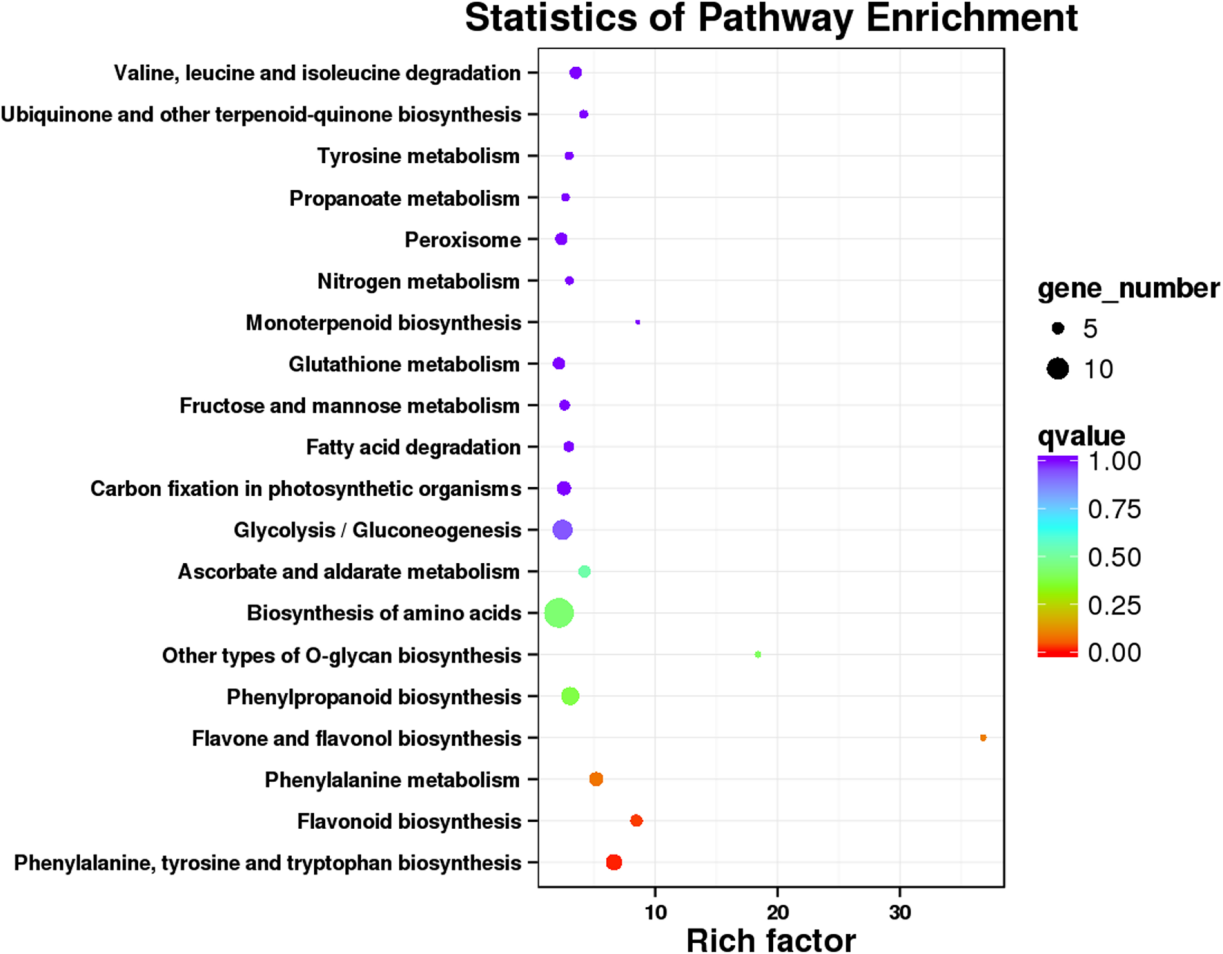
The top 20 KEGG pathways with the highest representation of differentially expressed genes (DEGs) in the B and D groups of plants (B vs. D).

**Figure 9:**
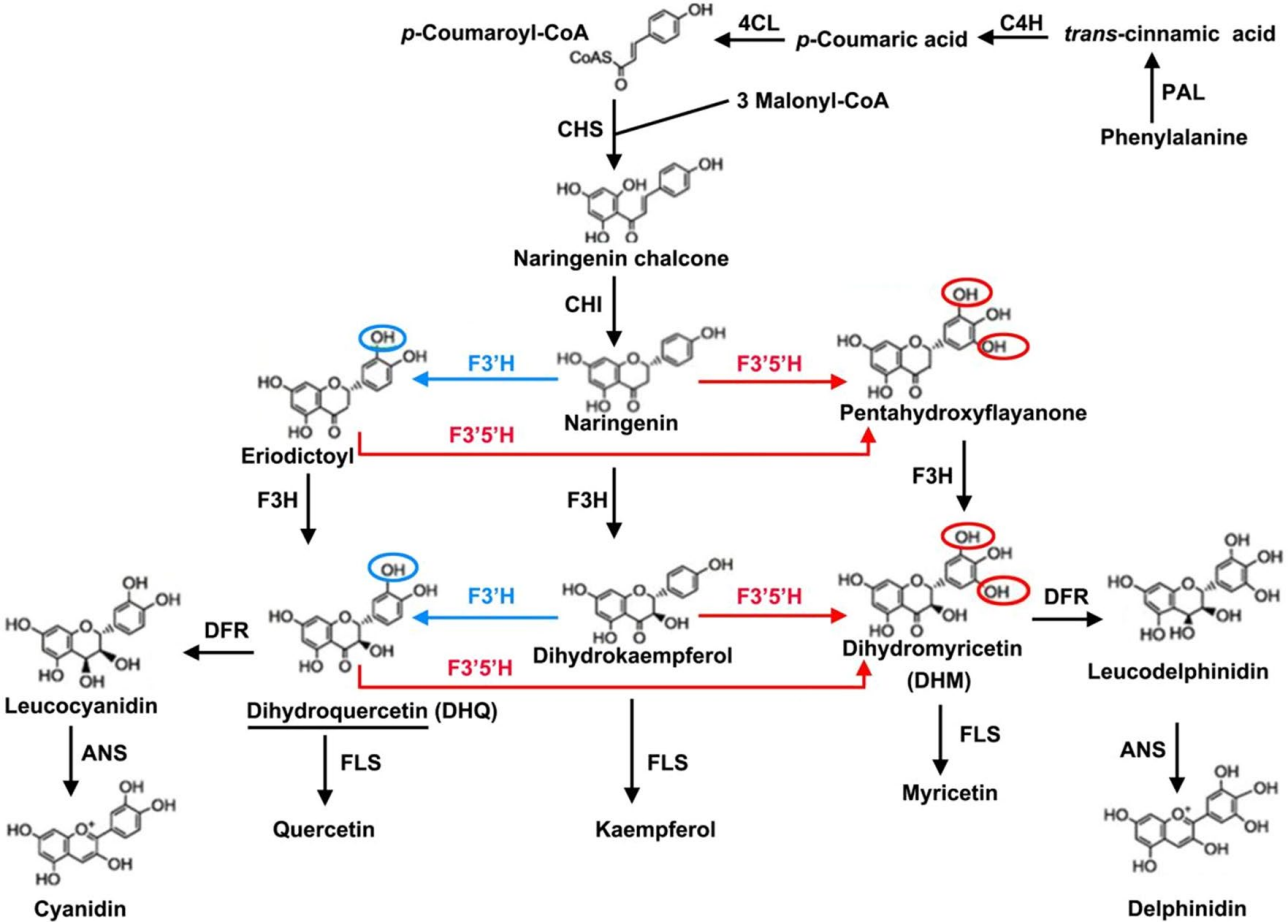
The main flavonoids biosynthesis pathway in *A. grossedentata*. The pathway was referenced to ko00940, ko00941, and ko00944 in the KEGG pathway. Abbreviations are as follows: PAL, phenylalanine ammonialyase; C4H, cinnamate 4‑ hydroxylase; 4CL, 4‑coumarate‑CoA ligase; CHS, chalcone synthase; CHI, chalcone isomerase; F3’H, flavonoid 3’‑ hydroxylase; F3’5’H, flavonoid 3’5’-hydroxylase; F3H, flavonoid 3‑hydroxylase; DFR, dihydroflavonol 4‑reductase; FLS, flavonol synthase; ANS, anthocyanidin synthase; DHM, dihydromyricetin. The single underlined portions represent dihydroquercetin (DHQ). The red lines representation of the reactions catalyzes by F3’5’H. The blue lines representation of the reactions catalyzes by F3’H.

Three metabolic pathways are known to be related to total flavonoids biosynthesis in plants^30^: the phenylpropanoid biosynthesis pathway (KEGG number: ko00940), flavonoid biosynthesis pathway (KEGG number: ko00941), and the flavone and flavonol biosynthesis pathway (KEGG number: ko00944)^45–47^. Accordingly, the top 20 KEGG enriched pathways we found also included these three processes (Fig. 7); specifically, we identified 338, 76, and 7 unigenes respectively in ko00940, ko00941, and ko00944 in the *A. grossedentata* transcriptome, whose corresponding number of DEGs were 8, 5, and 2 (Figs. 7, 8). Based on these three pathways, a simplified main flavonoids biosynthesis pathway including DHQ and DHM was constructed, which provides useful information of how flavonoid accumulates in *A. grossedentata* (Fig. 9). Flavonoids begin their synthesis via the phenylpropanoid pathway. The enzymes called phenylalanine ammonia lyase (PAL, 3 DEGs; Table S8), cinnamate 4-hydroxylase (C4H), coumarate-CoA ligase (4CL, 2 DEGs; Table S8), and chalcone synthase (CHS, 2 DEGs; Table S8) catalyze phenylalanine to form naringenin chalcone (Fig. 9). Chalcone isomerase (CHI) subsequently catalyzes the naringenin chalcone into naringenin, and the latter converted to dihydrokaempferol, eriodictoyl or pentahydroxyflayanone via catalysis by flavanone 3-hydroxylase (F3H), flavonoid 3’-hydroxylase (F3’H, 1 DEGs, Table S8) or flavonoid 3’, 5’-hydroxylase (F3’5’H, 1 DEGs, Table S8), respectively (Fig. 9). Then DHQ is synthesized by the enzyme F3H (it catalyzes the eriodictoyl) or by F3’H (it catalyzes the dihydrokaempferol) and DHM arises from DHQ, dihydrokaempferol, and pentahydroxyflayanone (Fig. 9). Flavonol synthase (FLS) or dihydroflavonol 4-reductase (DFR) catalyzes the conversion of DHQ or DHM, and anthocyanidin synthase (ANS, 1 DEGs; Table S7) catalyzes the synthesis of cyanidin or delphinidin (Fig. 8). In this proposed pathway, F3’5’H is the key enzyme operated to directly synthesize DHM from DHQ (Fig. 9).

The expression levels of genes participating in the flavonoid pathway are regulated by several transcription factors (TFs): in particular, the MYB, bHLH TFs, and WD40 proteins—also called the MYB-bHLH-WD40 heterotrimer—figure prominently in regulating genes in the flavonoid biosynthesis pathway^31, 48^. In our study, we distinguished five MYB, bHLH and WD40-related differentially-expressed TFs from the DEGs data (Table S7), including three MYB TFs, a probable bHLH TF, and a WD40 domain-containing protein (Table S8). All five TF genes were shown to be up-regulated (B vs. D) (Table S8). These proteins might form a complex involved in regulating flavonoid (DHQ and DHM) accumulation in *A. grossedentata*.

### Confirmation of differential gene expression by QRT-PCR

**Figure 10:**
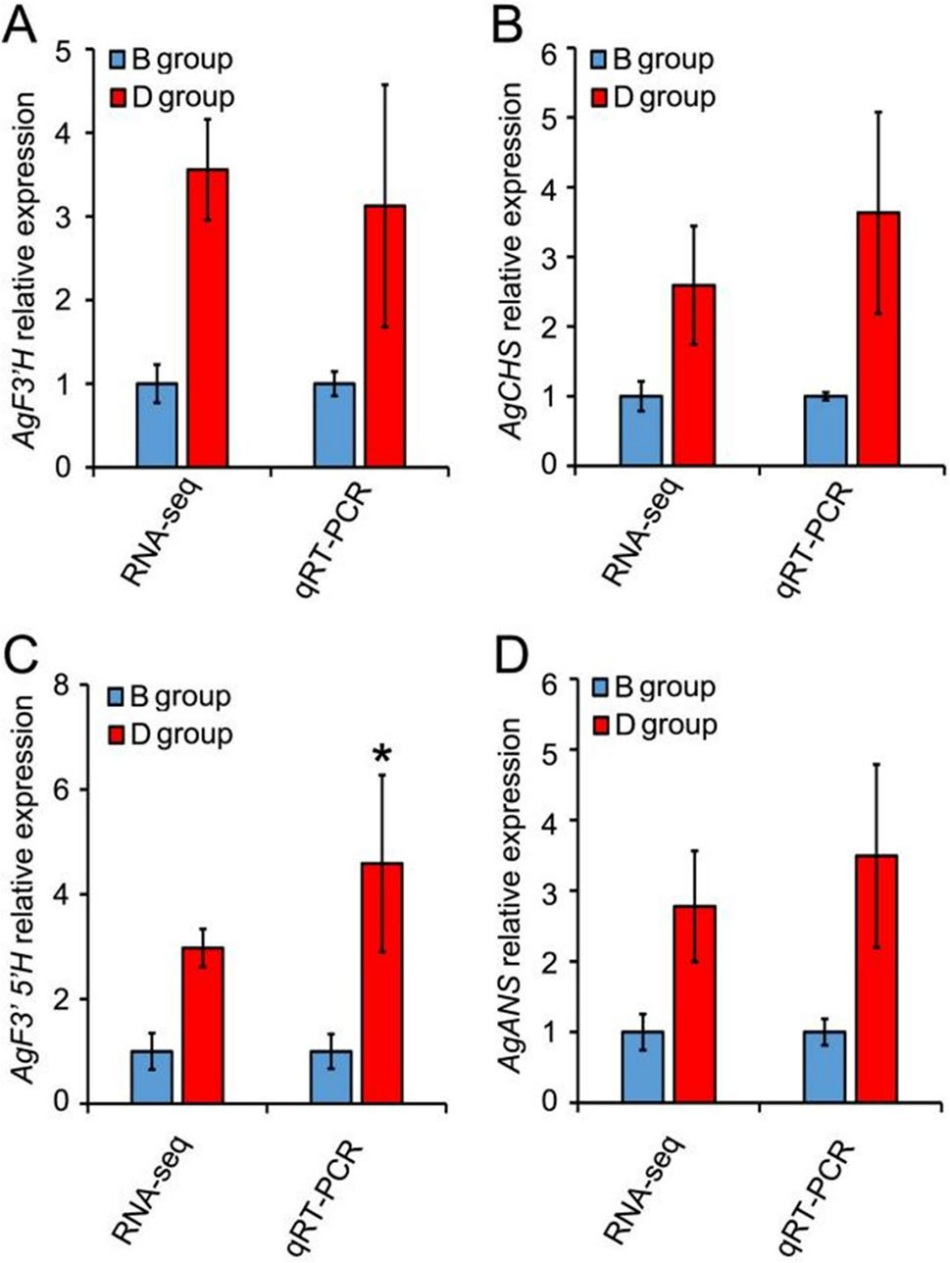
Comparison of mRNA accumulation of probable F3’H (**A**), CHS (**B**), F3’5’H (**C**), or ANS (**D**) genes in A. grossedentata between B and D groups of plants as determined by RNA-seq and QRT-PCR. For QRT-PCR analysis of gene expression, the expression of relevant genes was normalized to AgGAPDH’s expression level. Values are the mean ± SD. * P < 0.05, n = 3 (Student’s t-test). For the RNA-seq, we used the FPKM number to represent the expression abundance of each of the DEGs.

To determine the credibility of our RNA-seq data, the expressional profiles of four randomly selected genes related to DHQ and DHM accumulation were examined using QRT-PCR (Fig. 10). Their respective gene expression levels were normalized according to the *GAPDH* gene in *A. grossedentata*. As Figure 10 shows, the QRT-PCR validation of the four genes was consistent with the transcriptome sequencing data. Hence, our comparative transcriptome analysis was highly reliable for investigating relatively crucial genes involved in flavonoid (DHQ and DHM) accumulations in leaves of *A. grossedentata*.

## Discussion

### Candidate genes involved in *A. grossedentata* flavonoid (DHQ and DHM) biosynthesis

Flavonoids act as an important role in all living organisms. Understanding the flavonoid metabolic process is critical for the regulation of flavonoid-related phytochemicals’ (such as DHQ and DHM) biosynthesis and their potential applications in both food and medicine industries. Based on KEGG enrichment, we deduced a simplified main flavonoids biosynthesis pathway in *A. grossedentata* that includes DHQ and DHM (Fig. 9). The enzyme genes involved in this pathway may be assigned to two groups: one responsible for early flavonoid biosynthesis (encoding PAL, C4H, 4CL, CHS, CHI, F3H, F3’H, and F3’5’H) and the other for the late flavonoid biosynthesis (encoding FLS, DFR, and ANS). Structural enzymes (three PAL, two 4CL, two CHS, one F3’H, one F3’5’H, and one ANS) (Table S8) were differentially expressed between B and D group plants according to comparative transcriptome and QRT-PCR analysis (Fig. 10). PAL catalyzes the first step in the phenylpropanoid metabolic pathway. Often, more than one *PAL* gene exists in plant, and they are regulated by different environment stimuli^49^. In our data, three differentially-expressed *PAL* genes were all up-regulated in the D group (Table S8). The *4CL* is responsible for the synthesis of *p*-coumaroyl-CoA and, in our study, two *4CL* were differentially expressed: one up-regulated in the D group and the other gene down-regulated in the D group of plants (Table S8). The significantly down-regulated *4CL* gene may also be important, which belongs to the plant phenylpropane derivatives involved in the synthesis of flavonoids. *4CL,* catalyzing the production of differenttypes of plant resistance-related substances, is at the intersection in the metabolic pathway of phenylpropanoids to branching metabolic pathways such as flavonoids, lignin, and cinnamate^50^. CHS is encoded by more than one gene copy, and it is the key enzyme functioning before the synthesis of dihydrokaempferol^51^. Overexpression of *MaCHS* in transgenic tobacco plants markedly improved their environmental stress tolerance (to salt, drought, and heat) and modulated their metabolite profile^52^. Two *CHS* were significantly up-regulated in high-DHM *A. grossedentata* (Table S8). The high expression levels of *PAL*, *4CL*, and *CHS* should provide abundance substrates for synthesis of DHM (Fig. 9). Both F3’H and F3’5’H are key enzymes controlling the synthesis of anthocyanins and flower color^53^. In our study, one *F3’5’H* and one *F3’H* were up-regulated in the D versus B group (Table S8). This result suggested that high *F3’5’H* expression levels perhaps directly increase the production of DHM by using DHQ and dihydrokaempferol as the substrate (Fig. 9). Accordingly, we are most concerned with the F3’5’H enzyme because it directly converts DHQ to DHM (Fig. 9). Regulating the activity of *F3’5’H* may prove instrumental for changing the DHQ content in *A. grossedentata*.

The comparative transcriptome analysis between young and old leaves of *A. grossedentata*, which also on behalf of the high and low DHM materials, has been performed recently, and more transcripts involved in the flavonoid biosynthetic pathway of this plant was provided (including 13 *PAL*, 2 *C4H*, 2 *4CL*, 23 *CHS*, 5 *CHI*, 18 *F3H*, 10 *FLS*, 8 *F3’5’H*, 17 *DFR*, 1 *F3’H* and 2 *ANS*)^29^. This work is focus on annotation of the whole genes involved in high level of DHM production in *A. grossedentata* and giving us a lot of flavonoid related genes information; but the leaf samples they used stay in different growth stages, some genes may be influenced by the developmental regulation and display differential expression.Our reseach is focus on how to increasing the content of dihydroquercetin (DHQ) and diminishing the dihydromyricetin (DHM) accumulation in *A. grossedentata*. So, we used two different groups (contain differing amounts of DHM) (six independent individuals) of *A. grossedentata* in the same growth stage (eliminate the influence of plant growth and development). Our result more real shows the genes involved in flavonoid (DHM and DHQ) biosynthesis, especially the only one up-regulated *F3’5’H* gene which directly catalyzes the conversion from DHQ to DHM in the high DHM group and the other candidate genes are more useful for guiding the genetic breeding of high DHQ *A. grossedentata.*

### Candidate regulation genes (TFs) involved in *A. grossedentata* flavonoid (DHQ and DHM) Biosynthesis

The accumulation of flavonoids in plant tissues is affected not only by enzyme genes but also by several transcription factor (TF) genes. A transcription complex (MBW), containing MYB TF, bHLH TF, and WD40 protein, has been shown to regulate the expression of enzyme genes in the flavonoid pathway^31, 48^. Some R2R3 MYB transcription factors can activate *PAL* promoters to control the expression of this gene and the AtMYB transcription factor regulates the enzyme genes (*CHS*, *CHI*, and *F3H*) in the flavonoid pathway^54, 55^. Recently, two MYB TFs in peach fruit (MYB15 and MYBF1) were found to active the promoter of flavonoid biosynthesis genes, including *CHS*, *CHI*, *F3H*, and *FLS*^56^. The bHLH protein has a MYB- targeting region that enables their interaction; this interaction between bHLH and R2R3-MYB TFs is critical for the regulation of anthocyanin pigment biosynthesis in maize plants^57^. In the MBW complex, one WD40 protein could interact with different MYB proteins and thus play a key role by increasing the stability of the complex^58^.

Our results indicated that three, one, and one DEGs were predicted to code for MYB (MYB-like), bHLH, and WD40 proteins, respectively, in the DEG database (Table S7). Both of them were up-regulated in the D group (Table S8). These TFs and proteins may function alone or interact with each other to up-regulate the *PAL*, *4CL*, *CHS*, *F3’5’H*, and *F3’H*, consequently causing changes in DHM production in leaves. We had identified 1186 and 220 unigenes belong to “nucleic acid binding transcription factor activity” and “transcription factor activity, protein binding” according to GO enrichment, to which all eight DEGs were assigned (Fig. 6). However, many unigenes had no homologous matches in the protein databases and also lacked functional annotation. Although these genes have yet been identified in reported studies, some of them they may novel regulating genes in the flavonoid (DHQ and DHM) biosynthesis pathway.

### Increasing the content of DHQ and diminishing the DHM accumulation in *A. grossedentata* through genetic engineering

Flavonoid engineering has been performed in several organisms, including model and crop plants^59^. The content of anthocyanins (a type of flavonoid, pigment) is responsible for the different colors of flower or fruit and they have been linked to promoting human health. Therefore, anthocyanin enrichment was an early of flavonoid engineering in plants. Overexpression of *ZmDFR* in a petunia mutant led to increased anthocyanin content in the transgenic plants and finally changed flowers’ color^60^. Modulating the expression of genes for MYB and bHLH TFs from snapdragon in tomato plant clearly increased anthocyanins’ content of transgenic lines^61^. One ANS located downstream in the flavonoid pathway (Fig. 9) was up-regulated in our D plants (Table S8), perhaps driven so by the identified MYB or bHLH TFs (Table S8). High expression levels of *ANS* also implied anthocyanidins may occur at a constitutive high level in the D group (Fig. 1A). Recently, scientists created “Purple Endosperm Rice” by changing the expression of eight anthocyanin-related genes^62^. In floricultural plants, regulating the expression level of *F3’5’H* led to the production different flower colors^63^. We found a F3’5’H enzyme among our DEGs data, which directly converts DHQ to DHM (Fig. 9). A flavonoid 3’5’-hydroxylase (F3’5’H) (SlF3’5’H) enzyme from tomato relies on dihydrokaempferol or DHQ as a substrate^64^. Similar to SlF3’5’H, the *A. grossedentata* F3’5’H enzyme we identified also accepts dihydrokaempferol or DHQ as its substrate to produce DHM (Fig. 9).

Our data indicated the possible reason for why *A. grossedentata* accumulated abundant DHM (Fig. 1) yet a little DHQ is the plant’s high expression level of flavonoid (DHQ and DHM)-related genes, especially the up-regulated *F3’5’H* in the D group (Table S8, Fig. 9). Therefore, RNA interference (RNAi)-mediated silencing of the *F3’5’H* (Fig. 9) may offer one of strategies to increase the content of DHQ in *A. grossedentata*.

## Conclusion

*A. grossedentata* harbors an abundance of DHM yet a little DHQ. The biosynthesis pathway of DHM, DHQ and other flavonoids was unclear in this plant. In this article, candidate genes involved in flavonoid (DHM and DHQ) biosynthesis and regulation pathways have been elucidated using comparative transcriptome analysis of *A. grossedentata* containing extreme amounts of DHM under different genetic background and same developmental stage. Ten candidate enzyme genes and five TF genes associated with DHM and DHQ biosynthesis have been identified, including a key structural enzyme F3’5’H (directly catalyzes DHQ conversion to DHM). Our results deliver useful and important information for understanding flavonoids metabolic mechanisms of this potentially lucrative species. This work provides new insights into the molecular mechanisms that regulate flavonoids naturally occurring in *A. grossedentata*, especially DHM and DHQ. Furthermore, this work also suggests that it is possible to obtain new varieties of *A. grossedentata* with high DHQ content through gene knockout or silencing.

## Supplementary Information

Supplementary Tables.

Supplementary Table S7.
