# Supplementary material for: Exploring the genes involved in biosynthesis of dihydroquercetin and dihydromyricetin in Ampelopsis grossedentata
Source: Sci Rep. 2021 Aug 2;11:15596. doi: 10.1038/s41598-021-95071-x (PMC8329223; doi:10.1038/s41598-021-95071-x)
Supplement: Supplementary file 1 — Supplementary Tables. [file 41598_2021_95071_MOESM1_ESM.doc]

Table S1. List of Primers.

| Primer name | Primer sequence | Primer name | Primer sequence |
| --- | --- | --- | --- |
| 1F | TCTGATTCGATGAGGCTTGTG | 3R | CATTAATTTCTCTAACCGCCCG |
| 1R | AACATATGCACGGTCACTCC | 4F | AGGTTGCTGGAATGTAGTCG |
| 2F | CCTAAAGCCCGAGAAGTTACG | 4R | GGAGAAGTATGCTAATGACCAG |
| 2R | CATTCAAGTCCCTCACCCG | 5F | TTACTGACAAGGACAAGGCTG |
| 3F | CTAGTTCAACAGCTCGGACC | 5R | AGGAGCAAGGCAGTTTGTAG |

Table S2. The content of four compounds in *A. grossedentata*

| Sample ID | DHQ（mg/g） | DHM（mg/g） | Myricetin（mg/g） | Myricetrin（mg/g） |
| --- | --- | --- | --- | --- |
| B1 | 4.55 | 180.57 | 0.40 | 12.28 |
| B2 | 1.32 | 216.05 | 0.45 | 13.30 |
| B3 | 1.88 | 234.30 | 0.24 | 9.41 |
| D1 | 8.81 | 319.72 | 0.36 | 12.45 |
| D2 | 11.20 | 404.25 | 0.41 | 9.95 |
| D3 | 5.99 | 353.38 | 0.21 | 8.43 |

*Table S3. Correlation analysis between the content of four compounds*

|  | DHQ | DHM | Myricetin | Myricetrin |
| --- | --- | --- | --- | --- |
| DHQ | 1 |  |  |  |
| DHM | .825* | 1 |  |  |
| Myricetin | .131 | -.198 | 1 |  |
| Myricetrin | -.197 | -.538 | .810 | 1 |

* means P＜0.05

Table S4. Summary of sequencing quality and assembly data.

| Sample ID | Clean Reads | Clean Base | GC (%) | ≥Q30(%) |
| --- | --- | --- | --- | --- |
| B1 | 29,147,205 | 8,727,505,706 | 46.24 | 93.90 |
| B2 | 28,033,653 | 8,391,728,676 | 46.45 | 94.31 |
| B3 | 27,935,824 | 8,352,617,966 | 47.30 | 94.44 |
| D1 | 27,286,713 | 8,159,247,094 | 46.35 | 93.90 |
| D2 | 27,867,740 | 8,330,313,234 | 46.24 | 93.97 |
| D3 | 27,144,462 | 8,115,720,170 | 46.12 | 93.80 |

Table S5. Statistics of length distribution of unigene library

| Unigene Length (bp) | Total Unigene Number | Percentage (%) |
| --- | --- | --- |
| 200-300 | 26166 | 26.01 |
| 300-500 | 31061 | 30.88 |
| 500-1000 | 21652 | 21.53 |
| 1000-2000 | 11460 | 11.39 |
| 2000+ | 10245 | 10.19 |
| Total Number | 100584 |  |
| Total Length (bp) | 83624169 |  |
| N50 Length (bp) | 1489 |  |
| Mean Length (bp) | 831.39 |  |

Table S6. Statistics of annotation analysis of unigenes.

| Annodated databases | Unigene | Percentage (%) | 300<=length | length>=1,000 |
| --- | --- | --- | --- | --- |
| COG | 14876 | 26.09 | 5994 | 6219 |
| GO | 42682 | 74.86 | 20067 | 13331 |
| KEGG | 21197 | 37.18 | 10152 | 6327 |
| KOG | 29881 | 52.41 | 13587 | 10070 |
| Swiss-Prot | 35776 | 62.75 | 16303 | 12531 |
| Nr | 56003 | 98.22 | 26279 | 17392 |
| Pfam | 34365 | 60.27 | 13119 | 16470 |
| All | 57016 | 100 | 26629 | 17444 |

Table S8. The DEGs assigned to the flavonoid biosynthesis and regulation.

| Gene ID | Enzyme/Gene | Regulated |
| --- | --- | --- |
| c48392.graph_c0 | Flavonoid 3′-hydroxylase (F3′H) | Up |
| c52962.graph_c0 | Flavonoid 3′5′-hydroxylase (F3′5′H) | Up |
| c53486.graph_c2 | Anthocyanidin synthase (ANS) | Up |
| c52193.graph_c2 | Chalcone synthase 2 (CHS) | Up |
| c55275.graph_c0 | Chalcone synthase (CHS) | Up |
| c47575.graph_c0 | Phenylalanine ammonia-lyase 15, partial (PAL) | Up |
| c47575.graph_c1 | Phenylalanine ammonia-lyase 1-like (PAL) | Up |
| c59041.graph_c0 | Phenylalanine ammonia-lyase (PAL) | Up |
| c56901.graph_c1 | 4-coumarate-CoA ligase-like 6 (4CL) | Down |
| c58087.graph_c0 | 4-coumarate-CoA ligase 2 (4CL) | Up |
| c47454.graph_c0 | Transcription factor MYB34 | Up |
| c49493.graph_c0 | Myb-related protein 308-like | Up |
| c56640.graph_c2 | MYBPA1 protein isoform X1 | Up |
| c55100.graph_c0 | Basic helix-loop-helix protein | Up |
| c58327.graph_c1 | WD repeat-containing protein LWD1 | Up |
